# Pinpointing the politics of passing away. An empirical ethics case study on legislating assisted dying

**DOI:** 10.1186/s12910-026-01482-8

**Published:** 2026-05-14

**Authors:** Meike Gerber, Julia Fischer

**Affiliations:** 1 Institute for the History of Medicine, Faculty of Medicine Carl Gustav Carus, Dresden University of Technology, Dresden, Germany; 2 Department for Ethics and Law in Medicine, University of Vienna & Medical University of Vienna, Vienna, Austria; 3 Institute for Ethics, Collections and History of Medicine – Josephinum, Medical University of Vienna, Vienna, Austria

**Keywords:** assisted dying, assisted suicide, legislative debates, Germany, qualitative research methods, principlism

## Abstract

**Background:**

While the number of countries that have introduced or are trying to introduce statutory laws regulating the procedure of assisted dying is increasing, research has yet to address why some legislative attempts succeed and others fail. In Germany, which set out to regulate assisted suicide with an autonomy-based approach, two draft bills were unable to secure a parliamentary majority. This paper sets out to explain the underlying value disputes that contributed to the legislative failure.

**Methods:**

We conducted an interpretative analysis of 108 speeches and 18 declarations to the votes delivered in four parliamentary debates and 44 contributions from parliamentarians in the expert hearing in the Committee on Legal Affairs in Germany. For analysis of the publicly available stenographic protocols of the debates, we used MAXQDA Analytics Pro. Using thematic analysis, we aimed to capture the underlying value disputes as the core ideas driving politicians’ statements to explain why the attempt at regulating assisted dying failed in Germany.

**Results:**

Our analysis revealed that parliamentarians drew on beneficence-based reasoning when invoking the concept of autonomy. However, we identified two distinct camps that differ in how they interweave the concepts of autonomy and beneficence. Parliamentarians disagreed over which group is at greater risk of harm from inadequate assisted suicide legislation – eligible individuals denied access or ineligible individuals granted access. This determined whether they favoured a low-threshold or a more high-threshold approach toward regulating assisted suicide.

**Discussion:**

By disagreeing over which group is at greater risk of harm from assisted dying legislation, German parliamentarians engaged in a discussion style characterized as a debate over false negatives and false positives. At present, scholarship offers no definitive basis for alleviating parliamentarians’ concerns about the risks of either false negatives or false positives. Our analysis underscores that scholarship should engage with ethical reasoning and guide assisted dying policy by offering empirical insights into regulatory options that need to operate with accepting certain risks.

**Trial registration:**

Not applicable.

## Introduction

In Germany, to date legislative efforts to establish a statute regulating assisted dying have failed. The legislative process followed a landmark decision on assisted suicide (not voluntary euthanasia, which remains prohibited as killing on request) taken by the Federal Constitutional Court in 2020. The court ruled that the right to a self-determined death includes the right to make use of assistance provided by third parties for the purpose of taking one’s own life. According to the ruling, this right is “not limited to situations defined by external causes […], to serious or incurable illness, nor does it apply only in certain stages of life or illness” [1]. Consequently, German parliamentarians were required to establish assisted suicide legislation that aligns with what the literature calls an *autonomy-based model* of regulation [2]. This approach makes assisted dying accessible for everybody with decisional capacity, opening the possibility of assisted dying to people who consider their life as completed [3].

Bound by the 2020 ruling, the German parliament (*Bundestag*) began work on standardising the process of assisted suicide. Following initial debates on orientation, several draft bills were introduced, debated intensively among parliamentarians and experts, and ultimately narrowed down to two final proposals. The eligibility requirements were the same in both draft bills: the requesting person must be of legal age and, above all, their request for assisted suicide must be autonomous. That means that no acute mental illness may influence the formation of will, and the decision to commit suicide must be voluntary (without external pressure), permanent, and made with full knowledge of the alternatives and consequences. However, the draft bills differed considerably in terms of their safeguards and the principles governing access to assisted suicide. For instance, in the more restrictive bill, it was up to the individuals seeking assisted suicide to find physicians willing to support their request, and the physician confirming the free will of the requesting individual must hold certification in psychiatry. In contrast, the more liberal bill aimed to establish state-recognised and low-threshold counselling centres to provide open-ended counselling to any individual seeking assisted suicide. In July 2023, the parliamentary vote was cast. 589 out of 736 parliamentarians voted in favour of at least one of the two draft bills. However, no draft bills received a simple majority which would have been necessary for a draft bill to pass. Thus, both draft bills failed. As a result, at present assisted suicide is carried out in Germany in the absence of comprehensive legal regulation governing its process, with controversial practical implications [4–8].

To date, scholars have not yet examined why the German legislative process failed. Political theory suggests that legislative processes often fail due to value disputes [9]. When legislative processes involve competing normative commitments, lawmakers are faced with disagreements that cannot be resolved simply through technical adjustments or bargaining over material interests. Value conflicts tend to be deeply embedded in ideological identities, limiting the willingness to engage in pragmatic problem-solving. As a result, deliberation stalls, coalition-building becomes fragmented, and even well-designed institutional procedures are often unable to bridge incompatible worldviews [10]. Assisted dying is a profoundly normative issue and it can be assumed that this dynamic was at play in the German context. Yet many other countries have successfully passed assisted dying legislation. The number of countries that have succeeded in adopting statutory laws regulating the procedure of assisted dying has increased over the last decade and is expected to grow further [11–16] for an alternative reading. Relevant laws exist in Austria, Belgium, Canada, Luxembourg, the Netherlands, Portugal, Spain, several US states (including Oregon, Washington, Montana, Vermont, California) and several Australian states (such as Victoria, Western Australia and Tasmania). However, none of these countries adopted an autonomy-based approach to regulating assisted dying [2, 17, 18]. While not all laws explicitly mention suffering [19], they nonetheless adopt what the literature categorises as a *joint view*, linking access to assisted dying to decisional capacity but also to suffering from a medical condition [18, 20]. In doing so, around the world laws standardising the process of assisted dying support a prominent position in medical ethics that argues that assisted dying may only be considered ethically justifiable when it serves the best interests of the person in the sense of alleviating current unbearable suffering or preventing future inevitable suffering [21–25].

To offer empirical insight that can help explain why the legislative process in Germany failed, we conducted an interpretive analysis of speeches delivered by parliamentarians during plenary sessions dedicated to draft bills regulating assisted suicide. We searched for the underlying value disputes that may have contributed to the failure of the process. Rather than focusing our analysis on autonomy, as the guiding value established by the German Federal Constitutional Court and thus required as the principle central to parliamentarians’ deliberations, we set out to identify which values might be positioned in contrast to autonomy. Our study aims to illuminate the normative tensions and underlying values at stake, and to explain why the legislative effort failed. More broadly, our objective is to contribute to the small but growing body of research that examines the crafting of laws on assisted dying [26–28]. The *politics of passing away* is an emerging field of policy-making and is pushing legislators into relatively unfamiliar territory, not least because in most countries assisted dying is still unlawful [12]. The lively ethical debate on legislating assisted dying must be accompanied by empirical studies that examine what is feasible in practice – in our case, how parliamentarians approach the task of implementing an autonomy-based approach. We agree with authors who problematise the fact that “we know very little about why some attempts to change the law fail and others succeed” [15]. Addressing this gap is essential because both ethical reasoning and empirical insight are needed to develop a regulatory framework for assisted dying that is coherent in theory and works in practice.

## Materials and methods

Our study focuses on the four parliamentary debates on assisted suicide held in the German Bundestag following the Federal Constitutional Court’s judgement described above, including an expert hearing in the Committee on Legal Affairs. The hearing counts as a place of political deliberation as here the draft bills were critically examined by legal, medical and ethical experts who answered parliamentarians’ questions. As the expert statements do not express value-based disputes of parliamentarians, our analysis included only parliamentarians’ contributions in the expert hearing, not the expert opinions themselves. Although these contributions mainly consisted of questions to experts, the questions were oftentimes framed with short statements and comments, showing certain tendencies in opinion. Thus, we included the hearing in our analysis as an instrument of parliamentary opinion making in which value-based disputes of parliamentarians find expression. In total, we analysed five documents covering 108 speeches, 18 declarations to the votes, and 44 contributions from parliamentarians in the expert hearing (see Table 1).

**Table 1:** List of analysed documents collected via the online archives of the German parliament and the Committee on Legal Affairs

Document reference number	Date of the debate	Main focus	Analysed speeches	Analysed declarations to the votes	Analyzed parliamentarians’ contributions in expert hearing
19/223	21.04.2021	Agreed debate: Assisted suicide	42	n/a	n/a
20/36	18.05.2022	Agreed debate: Assisted suicide	27	n/a	n/a
20/45	24.06.2022	First consulting of draft bills	14	n/a	n/a
20/32	28.11.2022	Hearing in the Committee on Legal Affairs	n/a	n/a	44
20/115	06.07.2023	Second and third consulting of draft bills	25	18	n/a
Total number of analysed speeches, contributions and declarations			108	18	44

All documents are marked with an official reference number and are publicly available via the parliamentary archives, adding to the transparency of this study. We analysed the stenographic protocols of the debates and the expert hearing using MAXQDA Analytics Pro (Version 24.11). We conducted a thematic analysis, a qualitative interpretative method suited to capturing the core ideas and meanings that unite a set of observations by supporting a deep understanding of the material [29–31]. That said, we were aware of other classifications of arguments used during legislative debates on assisted dying that used interpretative methods to summarise data topics [26, 27]. However, our research interest was not focused on a descriptive mapping of all the arguments that politicians used, but rather in arriving at a meaning-based interpretation of the material.

Using thematic analysis [32], we were able to focus on capturing underlying value disputes as the core ideas driving politicians’ statements in order to explain the unique dynamic of the German debate: as the discussion was pre-centred on autonomy, in accordance with the Federal Constitutional Court ruling, and yet still no regulation was reached, our aim was to explain why some attempts at regulating assisted dying succeed and some fail. Therefore, we analysed the speeches to examine *how* politicians refer to autonomy and other values as a means of justifying their approach to regulating assisted dying compared to other approaches taken in an autonomy-based framework.

The data interpretation was performed in German to ensure precise analysis. The quotes were subsequently translated by a professional translator. The first author performed the full analysis, with both authors reviewing the coding structure and internal stringency of the initial classification and modifications. After reading and becoming familiar with the data, the first author generated initial codes in a systematic fashion. In the next step, these codes were collated into potential themes. The initial classification was determined by analysing the second and third debates on two draft bills in July 2023: this covered not only the most recent debate, but also the one in which legal regulation failed, making it the most important for our research interest. Initial analysis identified five themes: (1) autonomy as justification for high-threshold regulation, (2) autonomy as justification for low-threshold regulation, (3) beneficence as justification for restriction, (4) beneficence as justification for low-threshold regulation, and (5) the role of the state. While applying our categorisation to other debates and the expert hearing, we coordinated iterative refinements by checking if the themes work in relation to coded key extracts. During the process, we found that, rather than being treated as separate entities, references to beneficence and autonomy were linked in two different ways, and shaped the key argument of the politicians’ speeches. Further collation left us with a thematic map of two main camps, each with a value position defined by how they interweave the concepts of autonomy and beneficence: “autonomy and beneficence as justification for high-threshold regulation” and “autonomy and beneficence as justification for low-threshold regulation”.

## Results

The value of autonomy plays a central role in the parliamentary debate in the German Bundestag on the regulation of assisted suicide. Interestingly, so does the value of beneficence. Our analysis revealed that members of the Bundestag draw on beneficence-based reasoning when invoking the concept of autonomy. Among those parliamentarians who constructively engaged in the legislative process, we identified two distinct camps that differ in how they interweave the concepts of autonomy and beneficence – that is, in the types of arguments they employ and how these are linked. While those parliamentarians who engaged constructively in the debate argued in favour of one of the draft bills, a few parliamentarians did not support either bill. Those who argued against both draft bills only joined the debate to express their dissatisfaction. Dissatisfaction came in two ways. There were parliamentarians who argued that more public debate is needed and further work on drafting bills is required before any draft bill may be put to vote. In addition, there were also parliamentarians who explicitly announced not to support either bill because of general concerns regarding assisted suicide and its legislative regulation. However, our analysis revealed that the central conflict occurred between two camps, one of which supported a more liberal approach towards assisted suicide, while the other argued in favour of restrictive access. The central conflict was not about whether assisted suicide should be banned or legalized. The central conflict was about how to regulate assisted suicide via legislation. The following section thus focuses on introducing the two camps as outlined before, and their respective lines of reasoning.

### First camp: autonomy and beneficence as justification for low-threshold regulation

The reference to autonomy occupies a central position in the parliamentarians’ speeches. However, it is striking that the emphasis on autonomy is often embedded in beneficence-based arguments, or beneficence is a significant part of their argument. In this camp, beneficence is understood, above all, as facilitating justified assisted suicide. Firstly, it is argued that the protection of life must not be given precedence over self-determination.

Every individual has a right to a self-determined death as well as the right to seek assistance in exercising this right. Every self-determined individual is allowed to arrange their own death in a manner they deem worthy. Life cannot and must not be protected where this contravenes the autonomy of the individual [33].

Parliamentarians in this camp largely based their arguments on the conviction that making assisted suicide accessible is an act of beneficence. This is expressed through arguments that we have coded with ‘do not leave suffering people alone’ or ‘a lack of legal certainty leaves those affected alone’.

We feel it particularly important not to leave people alone again – alone with their pain, their fear and their desire to be allowed to die. That is why we need a legally admissible solution that everyone is confident will be accepted as valid by the Federal Constitutional Court. That is why we must not return to threatening criminal proceedings. That is why we need structures that are immediately accessible to everyone, without months of waiting for specialist appointments. Not only that, but the only way to help people who have veered off the path is to provide proper access [33].

Beneficence-based arguments are key here: they are the relevant motivator and decisive argument. It is true that, in the middle of their speech, the parliamentarian emphasises the importance of autonomy in the sense described above. However, the decisive arguments that suffering people should not be left alone, and that a law has the task of making it easier for them to access assisted suicide in a difficult situation, support the conviction that it is an act of beneficence to enable justified assisted suicide.

According to another decisive argument presented by proponents of the first camp, low-threshold access to assisted suicide could improve quality of life. This is achieved by reducing the fear of being forced to endure unbearable suffering, with the result that death itself becomes less threatening.

It is likely that we all harbour the wish that the actual process of dying will not involve suffering. Some people approach dying with humility; but most would rather live with the expectation of having the option to decide for themselves, and to act as they see fit. This hope can serve to effectively reduce fear and feelings of powerlessness. Dying becomes less threatening. It’s certainly easier to live with, and that’s why, for me, assisted suicide is also support for life [34].

This excerpt illustrates the close connection between arguments based on beneficence and autonomy in the liberal camp: although self-determination at the end of life is hugely important, it is not seen as an end in itself, but as a condition for improving the quality of life. The primary intention behind enabling justified assisted suicide is a qualitative improvement of life and death, achieved by reducing fear and making death less threatening.

Although proponents of the first camp vary the degree to which they base their argumentation on beneficence considerations, justifying assisted suicide purely for reasons of individual self-determination without reference to beneficence-based arguments such as release from suffering is found only extremely sporadically in this camp. Instead, self-determination and the suffering of the individual are combined in a form that allows the concepts of autonomy and beneficence to merge almost completely: the social expectation that suffering should be accepted contradicts the principle of autonomy. This must be reversed: the expectation of exercising autonomy (to die in a self-determined manner) establishes the right to either accept or to end the torment of suffering.

I am always surprised that there is constant talk of social pressure, but some who use that argument always think that it exists only in one direction. There can also be social pressure in the opposite direction: that people are expected to put up with a situation which is distressing or agonising for longer […]. I have to speak up because I hear it again and again, and it is starting to annoy me [35].

### Second camp: autonomy and beneficence as justification for high-threshold regulation

In their speeches, representatives of the second camp place a particular focus on the danger of compromised autonomy. Accordingly, they argue for high-threshold access to assisted suicide under the conditions established in the far-reaching landmark ruling. They refer primarily to beneficence, to plead for the avoidance of assisted suicides that they consider questionable or wrong. This goes as far as some raising concerns regarding the option of assisted suicide in general. By referencing social pressures which endanger autonomy, they also define the category of assisted suicides perceived as questionable more broadly than representatives of the first camp.

Proponents of high-threshold access to assisted suicide make self-determination conditional on the state fulfilling its duty to protect life – especially at the end of life and in existential crises. In this context, the protection of life is cited not only as integral to but also a prerequisite for autonomy.

Today we see a rather dramatized conflict between the protection of life and health on the one hand, and civil liberties on the other. […] For me, the priority has always been clear: without the protection of life and health, there is no basis for freedom [34].

This second camp argues that self-determination is endangered by social and external pressures. This is especially true for vulnerable groups whose autonomy requires particular protection. By referencing social pressures that endanger autonomy, they argue for higher hurdles as a means of avoiding assisted suicides which are perceived as questionable. Accordingly, there is a moral responsibility which arises, above all, from the compromised autonomy of vulnerable groups and those wishing to commit suicide.

Self-determination cannot function without protection of the weak. Respect for life demands this. I don’t want people in nursing homes to be put under pressure – anyone who has ever experienced this knows what it’s like – to end their lives because of this pressure, that assisted suicide would be a supposedly simple, good, painless, quick solution. I want everyone in these situations to get all the protection and help they need [36].

In this context, it is argued that society has a moral responsibility to turn towards suffering individuals with beneficial care.

That’s why I’m worried. I worry about the lonely, about those left behind, about those who think they are only a burden to others, about those who ask themselves: Is my life still worth anything? Dear Colleagues, people must answer these questions for themselves. But as a society, we should call out to them: Yes, your life is of value. Every life in this country is valuable [33].

Representatives of the second camp emphasize the importance of ensuring that every individual experiences their life as valuable—to themselves and to society. They understand the establishment and promotion of alternatives to assisted suicide, such as the provision of palliative care, as means of contributing to this goal. Suicide prevention is regarded as another such means. It is argued that society must clearly signal to everyone that their life is worth living; otherwise, individuals may feel pressured toward assisted suicide. According to representatives of the second camp, such pressure undermines the possibility of a genuinely autonomous decision. By invoking beneficence-based arguments—for example, that society must not abandon people who are suffering—representatives of the second camp challenge the possibility of true autonomy more fundamentally than those in the first camp.

For representatives of this second camp, the primary role of a law regulating assisted suicide is to prevent questionable assisted suicides and thus to counteract the normalisation of assisted suicide. The fear of normalisation is the fear of (businesslike) assisted suicide becoming socially accepted as a “normal means” [37] of ending life, and at the same time of the pressure that vulnerable groups may feel as a result of this development being uncritically accepted. The narrative of normalisation is countered by the formula of ‘making possible without promoting’:

Just as it is right to make assisted suicide possible, it is also right to counteract the normalisation of suicide. […] Why? Because then there is also a danger to people’s self-determination, because then the pressure increases, so people begin to ask themselves: Is it still worth it? Am I not just a burden? How much am I costing? – These questions demand a rebuttal. Because no one in this country should feel superfluous. […] We want to be a good country for everyone. That is why I ask you: Join us in making assisted suicide possible without promoting it! I ask for your support [36].

In this context, some representatives of the second camp also refer to the slippery slope argument: that the regulation of assisted suicide, once established, will be extended to groups of people who were not originally envisaged – a development that the state must counteract.

An assisted suicide law that provides no or too little real protection will open the way to normalising assisted suicide; in neighbouring countries, too, the debate did not begin with the inclusion of dementia patients, those with depression, or children, but now this is reality. When it comes to the far-reaching question of whether one can justify the path to normalising suicide, each and every one of us must examine our own consciences. We should not be under any illusions here: it is not the question of what one thinks one wants, codified with catalogues of criteria or procedural regulations; what counts is what you actually get [36].

## Discussion

Our analysis shows that German parliamentarians place crucial importance on both the values of autonomy and beneficence in regulating assisted suicide. However, we found these values were interpreted in two ways, depending on whether parliamentarians favoured low-threshold or high-threshold access. Proponents of low-threshold access to assisted suicide do not see autonomy as an end in itself, but rather as a means to providing beneficial care for people who would otherwise be left suffering. Proponents of more restrictive access see autonomy compromised through social pressure, which could be further reinforced should assisted suicide become normalised. Therefore they pledge to counteract developments that endanger vulnerable people from experiencing their lives as not worth living and regard this opposition as beneficial. Irrespective of these contrasting interpretations, parliamentarians did not separate the two values but instead drew on beneficence-based reasoning when invoking the concept of autonomy. Neither camp’s line of argumentation was effective without the two values being linked. Therefore our findings suggest that the legislative process did not fail because the two camps disagreed about which value mattered more: autonomy or beneficence. Both camps portrayed themselves as valuing both principles. Instead, the legislative process failed because they could not agree on *who* required beneficence-based attention and *whose* autonomy was at stake. The conflict was therefore less about the importance of autonomy versus beneficence, and more about the application of both principles to different groups. Parliamentarians expressed different views on who needs beneficence-based attention for the purpose of developing autonomy. The high-threshold camp focuses on protecting vulnerable people from potential abuse. In contrast, the low-threshold camp is more concerned about people who, despite their explicit will, are denied access to assisted suicide where it would be justified, preventing them from ending their suffering. In summary, our findings indicate that parliamentarians were unable to agree on which group warranted greater concern and which faced a higher level of risk. The obstacle to the legislative process was therefore not a value dispute over autonomy versus beneficence, but rather a dispute over which groups and individuals are at greater risk.

By disagreeing over which group is at greater risk of harm from assisted dying legislation, German parliamentarians engaged in a discussion style that the welfare literature characterises as a debate over false negatives and false positives [38, 39]. In contexts where governments must decide how to regulate access to benefits or rights, debates frequently devolve into a battle between two camps: those who favour an inclusion-priority approach to regulation, and those who advocate for an exclusion-priority approach [40–42]. An inclusion-priority regulation – the first camp in the German parliamentary debates – focuses on eligible individuals and aims to ensure they are granted access. It seeks to prevent errors of exclusion that wrongly deny access to eligible individuals (meaning false negatives). In contrast, an exclusion-priority regulation – as favoured by the second camp – focuses on ineligible individuals: accepting a higher risk that some eligible individuals will be denied, it aims to restrict access to ineligible individuals to prevent inclusion errors where ineligible individuals are granted access (meaning false positives).

At present, scholarship offers no definitive basis for alleviating parliamentarians’ concerns about the risks of either false negatives or false positives. In the context of regulating assisted dying, a false negative occurs when an eligible person is unable to achieve assistance in dying because the regulatory framework organises the process in an overly restrictive or risk-averse manner. When safeguards are designed to be overly protective, they impede not only the individuals they aim to protect but also those for whom they were never intended. As a result, even eligible individuals may find their access to assisted dying hindered or effectively blocked. Authors stress that relevant laws must standardise the assisted dying process to ensure not only *de jure* but also *de facto* access [19, 43]. Attention is turning to the problem of overly complex, multi-step access processes which create barriers, especially for the severely ill who often lack the functional capacity to navigate them [44]. Authors thus conclude that it is untenable for a law to present assisted dying as an available option where the practical realities make it unattainable, thereby “making shambles” [45] of the very notion of choice the laws purport to uphold. These arguments are, however, theoretical in nature. While there are empirical studies problematising the wrongful denial of access to assisted dying [44, 46], they do not allow for the definitive conclusion that safeguards are *too* restrictive in the sense that eligible requesters are ultimately hindered from obtaining assistance in dying.

By contrast, a false positive in assisted dying would mean granting access to a person for whom it should never have been granted, such as a person who is being coerced or under socio-economic pressures [47]. According to some authors, several reports of MAID cases in Canada suggest the occurrence of false positives [48]. For example, one newspaper article refers to a woman with severe allergies who applied for assisted suicide because she could not afford to move to a low-allergen apartment, and another woman who was unable to pay for treatment for chronic pain because of overwhelming debt [49]. While there are authors who claim that laws or legal systems are already too permissive [50, 51], scientific evidence of the occurrence of false positives is inconclusive.

In general, assisted dying regulation, as well as assessment procedures for those requesting assisted dying, must operate with and accept a certain level of insecurity. This includes, for example, the power asymmetry and subjective element intrinsic to assessment procedures in which experts are free to refuse or grant assisted dying based on supposedly standardised criteria [52]. The expert, often a physician, undoubtedly has an influence over the interpretation and social acceptability of a dying wish [53, 54]. That said, risk assessments and legal regulations bring their own potential for paternalism, and new social insecurities that will always remain a ‘grey area’. This becomes particularly – but not exclusively – evident with regard to access for vulnerable groups and people with mental illness, together with the ambivalence and social embedding of dying wishes [55–57]. But even though scholarly discussion of regulations on assisted dying is aware that no system can be perfectly safe, this aspect often remains obscured as the literature continues to suggest that restricting parliamentary debate on evidence-based contributions will ultimately lead to the safest regulations [26, 27].

Drawing on our findings, we contend that political debates on assisted dying cannot be grounded in evidence alone, since the pivotal issue of false positives and false negatives is likely to remain irreducibly marked by a level of uncertainty. Instead of focusing on the alleged normative implications of inconclusive evidence, we propose that the limitations and ambiguity of such evidence be acknowledged. Our proposition, however, should not be taken to imply that scholarship and social scientific research in particular are unable to make a meaningful contribution. On the contrary, such research is well-positioned to address important epistemic gaps and to examine the extent to which the principal arguments on either side of the debate are borne out by existing experience. The production of more comprehensive empirical knowledge is therefore of considerable importance. The current scarcity of relevant evidence is likely to diminish over time, not least because several evaluation studies are presently underway to examine the implementation of newly enacted legislation in different jurisdictions [58–60]. In Germany, a study is investigating the current practice in the absence of a specific legal framework [61]. Insights generated by this emerging body of research will warrant careful consideration. This also underscores the importance of ensuring that legal frameworks remain sufficiently responsive to incorporate new empirical findings and to adapt to evolving practical developments.

Although scientific research holds considerable potential to generate insights that may improve the practice of assisted dying, it is important to remain mindful that some degree of uncertainty may be irreducible. Consequently, understanding the regulation of assisted dying as a policy problem means acknowledging the importance of both evidence as well as value-driven disputes. Here, the distinction between an inclusion- or exclusion-priority approach can shed light on a debate that is as much about values as it is about pragmatic and evidence-based policy-making in a situation where there will always be a certain level of insecurity. We propose accepting that state regulation of assisted dying will not be able to eliminate all relevant risk, but – and this is the critical point – it can be the foundation for critical public discourse [52]. Seen in this light, there are two key questions for a pluralistic democratic debate:

(1) why politicians and public are willing or unwilling to accept certain levels of risks when it comes to including or excluding people from access to assisted dying and.

(2) whether these risk assessments are backed by actual experience within a comparable regulatory framework.

Finally, the emphasis on vulnerable groups and socioeconomic factors, as shown in the German parliamentary debate as well as the lively public and academic debate in Canada [48, 62], probably indicates that values such as equality and justice are also relevant, and must be considered in addition to autonomy and beneficence [for two different approaches to an equality-based reading of assisted dying see 63, 64]. Regulating access based on the factors of inclusion and exclusion, i.e. simultaneously providing rights and ensuring protection, points to a different value dispute probably being of relevance for assisted dying regulation: the debate between equality and difference. Originally a classical feminist debate, the dispute around equality and difference centres around the question how justice is best served: by applying standards in a neutral manner to formally equal parties or if law made allowances for difference [65]. One could argue that following an autonomy-based approach regarding assisted dying solves this dispute in favour of the value of equality as there is equal access for everyone with decisional capacity. However, our results show, that questions of difference – for example regarding vulnerable groups, but also illness status, still matter in politicians’ perceptions of justice and in attempts at legal regulation. Nevertheless, examining this promising new value-based reading of parliamentary debates about assisted dying regulations in light of the justice dimension is beyond the scope of this article. Future research into the *politics of passing away* could benefit from applying this perspective, to move beyond the dichotomy of autonomy and beneficence and grasp the value-based disputes at the core of establishing – or hindering – assisted dying regulation. Here, it would be especially interesting to examine countries that did not initialise the legislation process on a primarily autonomy-based framework, and to identify the risk scenarios which were deliberated and consequently resulted in regulation.

## Conclusion

Our study revealed that German parliamentarians draw on beneficence-based reasoning when invoking the concept of autonomy. These findings challenge the concept of autonomy and beneficence as two distinct values in the regulation of assisted dying. By referring to welfare literature to classify the German debate as one about false negatives and false positives, our study succeeds in contributing to a better understanding of why some attempts at regulating assisted dying succeed and some fail: German parliamentarians differed in their assessment of which groups to be most concerned about – eligible people being denied access to assisted dying, or ineligible people being granted access. Consequently, they were unable to reach a consensus on thresholds. Therefore, the key question for pluralistic democratic debate is the risks politicians and the public are willing to accept when it comes to including or excluding people from securing access to assisted dying. Within this framework, it is important that scholarly discussion avoids upholding the illusion that assisted dying legislation can ever guarantee complete safety. Instead, putting the deliberation of risk scenarios at the core of assisted dying legislation can shed light on a debate that is as much about evidence-based policy-making as it is about values in a situation where there will always be a certain level of insecurity. Furthermore, when examining how politicians simultaneously navigate the conflicting priorities of granting rights while protecting them from unwanted outcomes, other values such as justice, and weighing of equality and difference, may be involved. This paper concludes by inviting scholars examining *the politics of passing away* to move beyond the dichotomy of autonomy and beneficence as the primary conflicting values in assisting dying regulation, and towards the deliberation of risk scenarios when it comes to including or excluding people from access to assisted dying.

## Data Availability

All data analysed during this study is publicly available via the parliamentary archives of the German Bundestag.
